# The epiphyseal tubercle in adolescent hips

**DOI:** 10.3109/17453670903153550

**Published:** 2009-08-01

**Authors:** Keith Tayton

**Affiliations:** Dept of Orthopaedics, Royal Gwent HospitalNewport, South WalesUK

## Abstract

**Background** It has already been shown in animals and using anatomical human specimens that chronic slippage of the adolescent upper (capital) femoral epiphysis is unlikely to take place by shearing due to the presence of an epiphyseal tubercle projecting down into the metaphysis.

**Material, results, and interpretation** Plain radiographs of 20 adolescents and CT scans of 9 of them were analyzed for evidence of the size and presence of this tubercle in vivo. These cases showed that CT scanography is the investigation of choice to illustrate this largely undescribed anatomical feature. They also illustrate the epiphyseal tubercle well, both in the anatomical position and at various stages of “epiphyseal slippage”, and confirm that it has a significant restraining effect on any tendency of the epiphysis to alter its position relative to the metaphysis.

## Introduction

Much has been written about slipped upper femoral epiphysis (SUFE), and the interested reader is directed to the excellent review written by Loder and his colleagues (Loder et al. 2000). Understandably, most of the literature is concerned with treatment and outcomes, and relatively little has been published on etiology or the mechanism of the slip.

SUFE is rare in animals, and it seems likely that this is due to a tuberosity projecting from the lower side of the capital epiphysis into the growth plate, and from there into an appropriately shaped socket in the metaphysis—which effectively prevents chronic slippage from taking place (Tayton 2007a,b).

I have found a similar but much smaller epiphyseal peg and socket arrangement in adolescent human femurs, and have demonstrated that small shearing-type slips of the epiphysis are unlikely to occur due to the configuration of the growth plate complex. I concluded that the most likely mechanism of slow chronic displacement is rotation of the epiphysis on the metaphysis (about the tuberosity), as a result of torque forces (Tayton 2007a,b).

I have also suggested that this rotatory displacement progresses until a point is reached when the epiphysis simply tilted off the metaphysis pulling its peg out of its socket (Tayton 2007 a,b)—a situation that would neatly explain the severe, acute-on-chronic slip. If correct, then these observations would have implications for clinical management.

In order to avoid needless radiation, I carried out a study on a small series of adolescents who were having radiographic imaging of their hips for reasons other than this project. The purpose of the study was to examine the images of the bone surfaces adjacent to the femoral capital growth plates, and in particular to see whether the epiphyseal tubercle could be visualized sufficiently well to allow an in vivo assessment of its size and hence of its stabilizing effect.

## Method

A consecutive series of plain radiographs of normal hips in 20 individuals aged 8–15 years was analyzed specifically to assess the size of the epiphyseal tubercle. Of these patients, 9 were also being investigated for hip pain and they underwent CT scanning. These scans were all traced and analyzed for size of the epiphyseal tubercle.

## Results

Initially, this study was designed to concentrate on plain radiographs of adolescent hips. However, the quality of definition of the capital growth plate was generally poor and most films were of limited value, with useful information being gathered largely by chance. Some did, however, show clear features that merit discussion.

**Figure 1.**
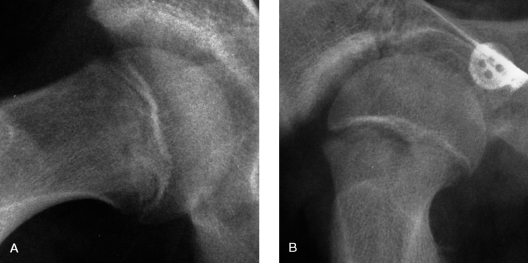
A. The right hip of a 13-year-old boy, showing a large epiphyseal tubercle. B. This is contrasted with the right hip of a 12-year-old boy, showing a fairly small epiphyseal tubercle, which nevertheless still has a stabilizing effect on the developing SUFE.

In the younger child the tubercle was rather wide and flat, and was noted to be somewhat similar to the findings in young lambs. The radiographs in Figure 1 show the epiphyseal tubercle in the adolescent to be a more pointed structure. Figure 1A shows a particularly well developed peg in the right hip of a 13-year-old boy who had all the clinical signs of a SUFE, and was subsequently shown on CT scanning to be suffering from a very early stage of the condition. Figure 1B illustrates a hip suffering from an early SUFE, and appears to be further confirmation that in later adolescence the epiphyseal tubercle becomes narrower, sharper, and more peg-shaped.

These films all show the restraining effect that the epiphyseal peg must have in SUFE, and they make it clear that any slipping of the epiphysis (in the usual meaning of the term) would involve significant dislocation of the epiphysis from the positions shown.

Anatomical variations are of course likely to be common, and one case was seen in a 14-year-old boy who appeared to have a double peg.

**Figure 2.**
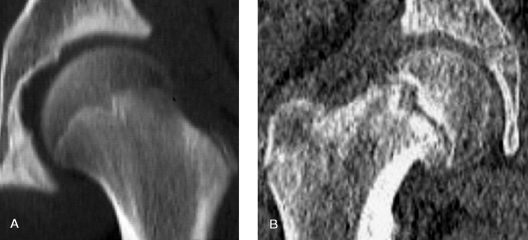
A. An AP CT scanogram of the left hip of a 14-year-old boy showing a well-developed epiphyseal peg. B. A similar CT scanogram of the right hip of a 13-year-old boy who has a poorly defined peg that appears to be showing signs of loosening.

**Figure 3.**
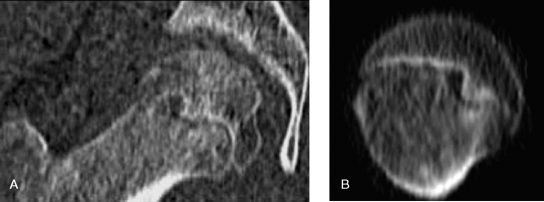
AP (A) and axial (B) CT scanograms of the right hip of the 13-year-old boy whose radiograph is shown in Figure 1A, now revealing an epiphyseal peg that seems to be a rather loose fit in the metaphyseal socket.

**Figure 4.**
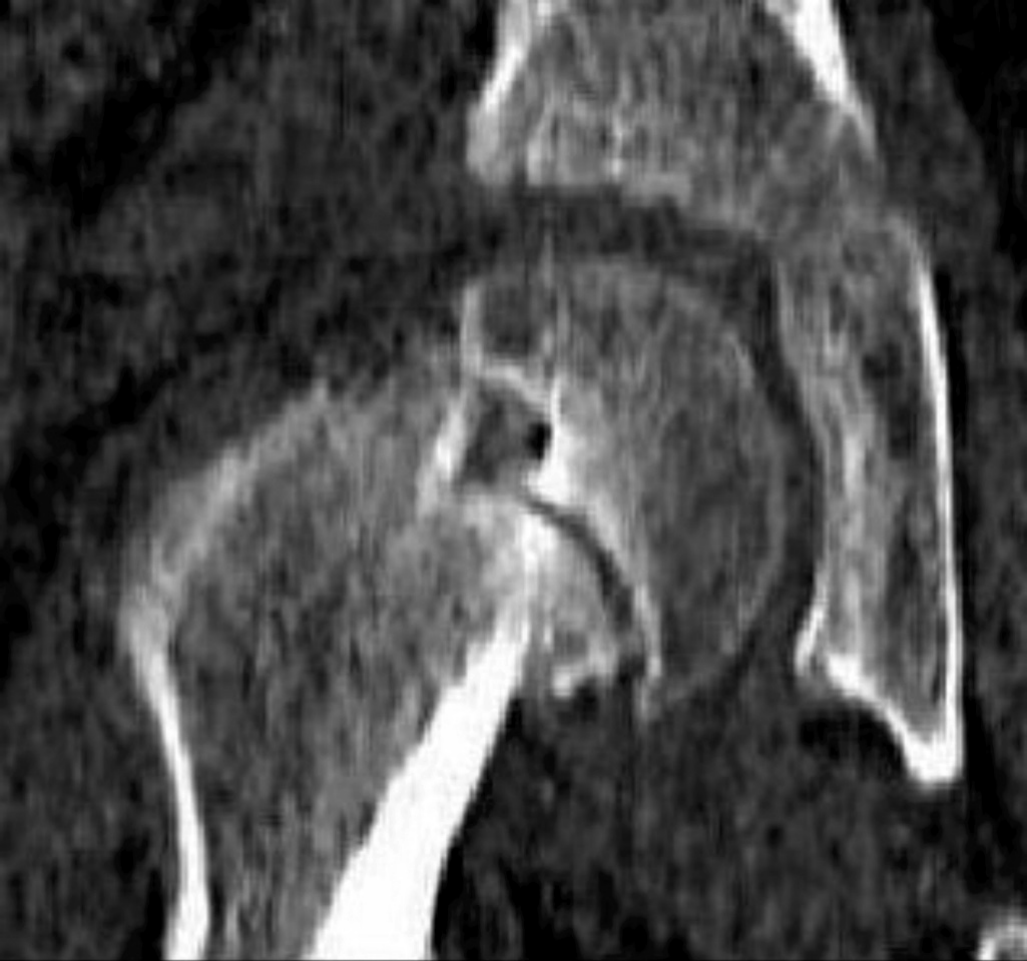
AP CT scanogram of the right hip of a 13-year-old boy, showing a well-developed epiphyseal tubercle that is clearly displacing out of the metaphyseal socket as an acute-on-chronic slip develops.

Of the 9 children with CT scans, only 4 had any diagnosed hip pathology—all being unilateral SUFE (Table). Figures 2, 3, and 4 are typical CT images, and show that the quality of delineation of the growth plate complex is far superior to other imaging techniques.

**Table:** The various children selected from routine orthopedic clinics

Study CT Scan	Sex of child	Age (years)	Unilateral slipped upper femoral epiphysis
	F	11	
	F	11	
	M	12	+
	M	13	+
	M	13	+
	F	13	+
	M	13	
	M	14	
	M	16	

Figure 2A illustrates the typical normal left hip of a 14-year-old boy showing a well-developed epiphyseal peg-like tubercle. A similar scan was obtained in a 16-year-old boy, which indicates that the peg probably remains an important feature until femoral bone growth has finished. Figure 2B is an AP CT scanogram of the right hip of a 13-year-old boy, which shows a well-developed metaphyseal socket particularly clearly—but on this cut, no epiphyseal peg. Some of the axial cuts did show the peg within the socket, however, and it is suggested that this image represents the very first stage of the rotational displacement of the epiphysis.

Figure 3 shows AP and axial CT scans of the right hip illustrated radiographically in Figure 1A, which again probably illustrates the very first stage of a SUFE, when the epiphyseal peg has just come loose in its socket. The young man had a 6-month history and all the symptoms and signs of an early SUFE. Interestingly, however, on the AP cuts the CT scan showed that although the epiphyseal peg seemed to be rather a loose fit within its metaphyseal socket, it looked otherwise normal. However, although the axial cuts also show the peg within the socket, it can be seen to be somewhat eccentrically placed and seems likely to be out of its normal position. The obvious explanation for this would be that the epiphysis has rotated out of position rather than slipped.

Finally, Figure 4 is an AP CT scanogram of the right hip of a 13-year-old boy with a SUFE. Once again, the epiphyseal peg is well shown but this time it is coming out of its metaphyseal socket and is not preventing the epiphysis from becoming displaced. This child had a history of several weeks of pain becoming much more severe recently, and clearly an acute-on-chronic SUFE is developing.

## Discussion

It is generally considered that SUFE occurs in susceptible growth plates exposed to appropriate shear forces, and many studies attempting to assess the resistance of growth plates to such forces have been carried out on both animal and human material (Chung et al. 1976, Peltonen et al. 1984).

A previously reported study by me (Tayton 2007b) confirmed the presence of the epiphyseal tubercle as a significant feature of the human capital growth plate complex, and a major obstacle to any slippage occurring as a result of shear forces (which for practical purposes act in a single plane). Unfortunately, the strength of the conclusions in that study was limited by the fact that there were only 11 anatomical specimens, several of which bore the erosive changes of aging.

The study reported here was carried out to extend the investigation, and in particular to try to assess the quality of the epiphyseal tubercle in vivo. With case numbers limited by ethical constraints, its findings confirm the proposition that the tubercle is a normal feature of the capital growth plate complex, although assessment of its size from the films was found to be difficult. In the previous study, it had averaged 4 mm in length on anatomical specimens.

Although it seems probable that this epiphyseal “peg” is present in most adolescents, it should be noted that the plain radiographs illustrated here have been selected for the clarity with which they show it, and that many were examined which failed to show any convincing sign of one.

An attempt to carry out this study using MRI scanning was abandoned because currently these scans give very small images and enlargement causes loss of definition due to pixel size. Within the limitations of pixel enlargement, well-developed epiphyseal tubercles could be seen in the younger child. On the other hand, excellent images of the epiphyseal peg were produced by CT scanning, which is clearly the investigation of choice.

The findings on the plain radiographs (and MRI scans) show that in younger children the tubercle is wide-based and fairly flat, and that as maturity progresses it becomes somewhat narrower, longer, and more pointed. As a result, its anti-slipping effect seems to change from one of friction at two irregular surfaces to one of pegging. The concept of pegging is supported by the CT scan studies on the older adolescents, where the tubercle was seen to remain reasonably prominent up to the age of 16. Of the 4 cases with SUFE who were CT-scanned, the tubercle in one was clearly acting as a pivot for the epiphysis, and in one other it was just beginning to lose good contact with the metaphysis—and it seems likely that it was twisting free of its socket.

Although the value of this radiographic study is restricted by the numbers, it does confirm the presence and size of the epiphyseal tubercle as a fairly regular feature in adolescents. It also shows that using CT scanning, the tubercle can nearly always be demonstrated and that it seems to be acting as a peg.

Tempting though it is to suggest that these findings represent the norm, a larger population survey is needed to establish this. The findings do, however, support the theory that a relatively stable chronic epiphyseal slip cannot take place in a simple linear direction due to the configuration of the bone surfaces adjacent to the growth plate, and in particular to the presence of the epiphyseal tubercle.

The clinical relevance of this is two-fold. Firstly, because in early chronic SUFE the epiphysis is reasonably stable, it should not be necessary to carry out an epiphyseodesis. The insertion of a properly anchored anti-rotation pin should be sufficient to control stability and so allow the physis to continue to grow normally and the femoral head to remodel. Secondly, in cases of unilateral slippage where the risk of a contralateral slip is considered high, a CT scan of the unaffected hip will indicate the size of its epiphyseal tubercle; and if it is dysplastic, then its prophylactic stabilization should be considered.
